# Idiopathic masseter muscle hypertrophy: a case report

**DOI:** 10.31744/einstein_journal/2019AI4506

**Published:** 2019-02-25

**Authors:** Joana Jorge Antunes, Susana Assunção Almeida, Ricardo Miguel Patrício de Carvalho Monteiro, Ana Mafalda Martins

**Affiliations:** 1 Hospital de Cascais Dr. José de Almeida, Alcabideche, Cascais, Portugal.

Idiopathic masseter muscle hypertrophy is a rare condition, characterized by unilateral or bilateral enlargement of this muscle, sometimes associated to mandibular angle exostosis.^(^ ^1^ ^)^ Its etiology is unknown, and there is a possible relation with unilateral masticatory activity, dental malocclusion, temporomandibular joint dysfunction, bruxism, or emotional alterations.^(^ ^2^ ^,^ ^3^ ^)^ The diagnosis is made primarily based on the clinical presentation, complemented with ultrasound and, if required, magnetic resonance imaging.^(^ ^3^ ^)^ It is important to make differential diagnoses with tumors or inflammatory processes in muscles, bones and salivary glands.^(^ ^2^ ^)^

**Figure 1:**
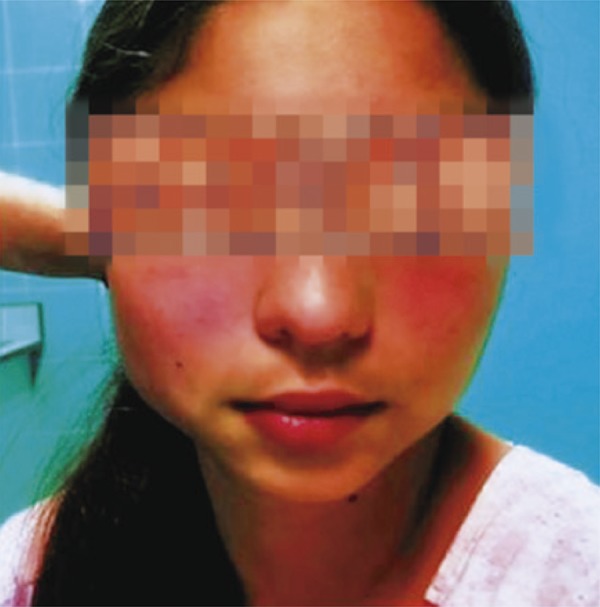
Adolescent with asymmetry due to right mandibular swelling

**Figure 2:**
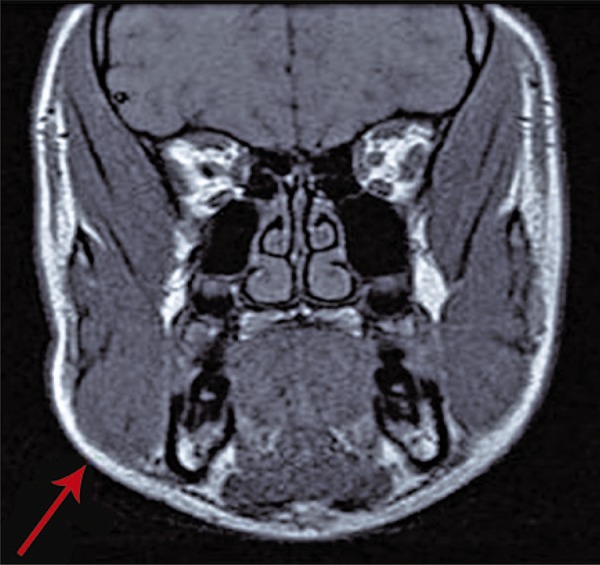
Coronal magnetic resonance imaging showing asymmetry of the right masseter muscle

We presented a case of a 15-year female adolescent, with no significant past medical history, seen at the Emergency Department, due to right mandible swelling, painless, which started three months before and presented progressive worsening. She had no past history of facial trauma, trismus, constitutional symptoms or periodontal disease. Upon examination (Figure 1), she presented facial asymmetry, swelling on the right mandibular angle, more evident with mandibular occlusion, and a mass was palpable, measuring 4cm in the longest axis, painless, with elastic consistency. There was an overlapping mass, with more consistent texture, 2cm diameter and attached to the bone. Laboratory tests showed no alterations, with negative inflammatory parameters and viral serology. Salivary glands ultrasound showed no detectable abnormalities. Face magnetic resonance imaging (Figure 2) demonstrated facial asymmetry involving the right masseter muscle and parotid gland, with no intrinsic pathology, and consistent with masseter muscle hypertrophy. The patient was referred to appointment at pediatric surgery, for aesthetic procedure, if necessary.

This condition has a benign behavior, with essentially aesthetic implications, because of asymmetric lower third of the face. The treatment is conservative, using some therapies, in selected cases, such as muscle relaxants and botulinum toxin, or by excising the excessive muscle fibers.^(^ ^2^ ^,^ ^4^ ^,^ ^5^ ^)^
